# “Young people, adult worries”: RCT of an internet-based self-support method “Feel the ViBe” for children, adolescents and young adults exposed to family violence, a study protocol

**DOI:** 10.1186/1471-2458-13-226

**Published:** 2013-03-15

**Authors:** Karin AWL van Rosmalen-Nooijens, Judith B Prins, Marianne Vergeer, Sylvie H Lo Fo Wong, Antoine LM Lagro-Janssen

**Affiliations:** 1 Department of Primary and Community Care, Gender & Women’s Health, Radboud University Nijmegen Medical Centre, PO Box 9101, Nijmegen, 6500HB, The Netherlands; 2 Department of Medical Psychology, Radboud University Nijmegen Medical Centre, PO Box 9101, Nijmegen, 6500HB, The Netherlands; 3 Department of Gynaecology and Obstetrics, Radboud University Nijmegen Medical Centre, PO Box 9101, Nijmegen, 6500HB, The Netherlands

**Keywords:** Family violence, Exposure to violence, Witness violence, Sexual and reproductive health, Children, Adolescents, Young adults, E-health, RCT, Mental health

## Abstract

**Background:**

Violence in families affects children. Exposure to violence is seen as child abuse. Figures show that about one third of children exposed to violence become victim or perpetrator in their adult life: known as intergenerational transmission. Violence also affects sexual and reproductive health. To prevent problems in adult life, children need help and support. However, while trying to protect their parents, children often do not seek help, or perceive the threshold as too high. Since almost all children of the current generation have access to the internet, an online intervention will make help better available for this target group. In 2011, an internet-based self-support method for children, adolescents and young adults exposed to family violence was developed in the Netherlands: “Feel the ViBe”. The intervention was developed in close collaboration with the target group. This article describes the protocol of the RCT to study the effectiveness of this intervention.

**Methods/design:**

This study is a randomized controlled trial using the method of minimization to randomize the participants in two parallel groups with a 1:1 allocation ratio, being an intervention group, having access to “Feel the ViBe” and usual care (UC), and a control group, having access to minimally enhanced usual care (mEUC) followed by access to the intervention after twelve weeks. Outcomes are measured with questionnaires on PTSD symptoms, mental health and sexual and reproductive health. Routine Outcome Measurement (ROM) will be used to measure a direct effect of participating in the intervention. Data from a web evaluation questionnaire (WEQ), user statistics and qualitative analysis of online data will be used to support the findings. To compare results Cohen’s d effect sizes will be used.

**Discussion:**

A RCT and process evaluation will test effectiveness and provide information of how the effects can be explained, how the intervention meets the expectation of participants and which possible barriers and facilitators for implementation exist. A qualitative analysis of the data will add information to interpret the quantitative data. This makes “Feel the ViBe” unique in its field.

**Trial registration:**

The Netherlands National Trial Register (NTR), trial ID NTR3692.

## Background

Violence in families mostly affects women and children. Cross-sectional surveys in General Practice show that the prevalence of family violence for women between 16–65 years of age is estimated at 30-41% [1-3]. The WHO defines family violence as any behaviour within an intimate relationship that causes physical, sexual or psychological harm, including acts of physical aggression, sexual coercion, psychological abuse and controlling behaviours. This definition covers violence by both current and former spouses and partners [4]. The Netherlands Youth Institute (NJI) includes in their definition that other family members, including children, are, directly or indirectly, affected by the exposure to the violence and considers it as child abuse [5]. In 2006, in 60% of the cases of family violence in Dutch women registered by the police, there were children living at home, and in most cases they were exposed to the violence against their mothers. This leads to an estimate of 15.340 children in the Netherlands exposed to family violence [6]. In 2008, 23% of the contacts with the Dutch Child Abuse Authority (AMK) concerned exposure to family violence [7].

Family violence contributes to significant morbidity, such as depression and anxiety disorders [8]. Children in families where violence occurs are in a very difficult position, often supporting the victim, trying to protect him or her, instead of being protected themselves. Their feelings and comprehension of ‘safety’ as well as their immediate safety are highly under pressure. Repeated subjection to violence and the interfamilial character increase the chance of developing mental health or behavioural problems. These children are as much at risk for long term negative consequences as children who are abused themselves. The consequences are diverse: mental health problems, such as affective and depressive disorders and suicide attempts, educational problems, such as school drop-out, behavioural problems, substance/drug abuse and risk taking behaviour. They also have a one-in-three chance of becoming either victim or abuser in their adult life. This is called intergenerational or transgenerational transmission [9-14]. Several theoretical models underlie these findings. Ehrensaft et al. and Carpenter & Stacks both give an extensive overview of these models, including the social learning theory of Bandura [11,15,16]. This theory states that children learn new behaviour by observing and imitating significant others, called modelling. If children are exposed to violence, they learn that violence is an acceptable or effective means of resolving conflicts with the partner. Other theories are the betrayal trauma theory [17] and the attachment theory: parenting stress can impact internalizing and externalizing behaviour and lead to increased stress and symptoms of posttraumatic stress disorder (PTSD) [18-20].

Family violence also influences reproductive and sexual health. A systematic review by Coker [21] addressed family violence and sexual health. Family violence was consistently associated with sexual risk taking, unplanned pregnancy or induced abortion, sexually transmitted disease (STD) and sexual dysfunction [21]. There is hardly any research studying the consequences of exposure to family violence on reproductive and sexual health issues in children and adolescents. The few studies focusing on adolescents exposed to violence at home, found an association with sexual risk behaviour, having sex before age 15, multiple partners, having a STD, unplanned pregnancy, and alcohol/drug use in relation to sexual activities [9,22-26].

In order to prevent intergenerational transmission, mental health problems and sexual/reproductive risk behaviour in adult life, it is important that early support for children, adolescents and young adults exposed to family violence is available. Support, however, is scarce. Most of the preventive interventions in the Netherlands are regional, do not offer specialized care or have a high threshold. Interventions specifically aimed at children and adolescents exposed to family violence are mostly group therapy. These interventions all require involvement and/or consent of parent(s), regular mental healthcare and/or need a referral from the GP or Child Protection agency. Knowing that these children often support their mother by trying to protect her, instead of being protected, they are not likely to seek help for themselves unless their mother is already receiving help [27,28]. Unfortunately, women who face family violence are often too afraid to seek help and mothers with children at home are even more vulnerable since they try to protect their children. Trying to maintain the family, they do not leave the abusive partner in many cases. Above that, regular health services frequently fail to deliver appropriate support due to long waiting lists and because they do not meet women’s needs [29,30]. The surroundings, abusers, mothers and GP’s are in most cases not aware of the long-term consequences of exposure to family violence for children [3]. Therefore, children and adolescents exposed to family violence are difficult to reach. Moreover, evidence is lacking on the effects of preventive interventions and the possible role of primary health care [31].

To prevent intergenerational transmission and negative effects on reproductive and mental health, a low-threshold support method is needed for children, adolescents and young adults exposed to family violence. Children and adolescents of the current generation often rely on the internet as source of information and to maintain their social contacts. In the Netherlands, 93% of the children age 6–18 use the internet and 78% use social networking sites. Of the adolescents, age 12–18, 96% have a mobile phone. After contacting friends in real life, the internet and their mobile phone are the most important sources for adolescents age 12–18. A good website according to adolescents age 12–18, gives them reliable information, provides social support and is safe [32,33]. Considering this, it is to be expected that children who are exposed to family violence will search for information on the internet. Although general information on sexual and reproductive health and general help, for example the ‘Kindertelefoon’ (‘Children’s telephone’: support for children by phone and chat), is available online, there is hardly any specific information on what to do. An internet-based self-support method can be a low-threshold method to give support. E-health is still a commencing method and most interventions available online are based on assumptions and literature instead of including the wishes, needs and demands of the target group. Because of constraints from ethical boards when it concerns minors, most of these interventions are not evaluated well. Peer support and peer education, however, are researched extensively and are nowadays recognized as effective methods to change behaviour [34-37]. Furthermore, social support has proven to be effective in adults exposed to violence and is associated with good mental and physical health outcomes [38,39].

In 2011 “Young People, Adult worries” started with the development of a new internet-based self-support method for children, adolescents and young people exposed to family violence. Based on opinions from the target group, experts and literature, “Feel the ViBe (Violence Beaten)” was developed. “Feel the ViBe” is intended as a freely available, low-threshold stand-alone intervention for children, adolescents and young people who are exposed to family violence. The primary goals are to provide (peer)support and information. If “Feel the ViBe” is successful, this may have large impact on the traditional healthcare, possibly leading to more help offered and less costs.

This study protocol briefly describes the development of the intervention, which took place in 2011, followed by the protocol for the effectiveness study.

### Primary objective

•To study the effectiveness of the internet-based self-support method “Feel the ViBe”.

### Secondary objectives

•To explore knowledge about sexual and reproductive health in children, adolescents and young adults exposed to family violence.

•To explore sexual risk taking behaviour in children, adolescents and young adults exposed to family violence.

## Methods/design

### Trial design

This study is a randomized controlled trial using the method of minimization, as described by Taves (1974), to randomize the participants in two parallel groups with a 1:1 allocation ratio, being an intervention group, having access to “Feel the ViBe” + usual care (UC), and a control group, having access to minimally enhanced usual care (mEUC) [40,41]. Subcategories for the process of minimization will be sex (male or female) and age (12–17 years old and 18–25 years old). The study is conducted in the Netherlands. This trial is registered in The Netherlands National Trial Register (NTR) and assigned the trial ID NTR3692.

### Participants

For reading purposes, we will use ‘adolescent’ from now on to refer to the target group. The definition of ‘adolescent’ for this study protocol is: children, adolescents and young adults in the age of 12 to 25 years old. The study population consists of two age groups: 12–17 years old and 18–25 years old. We chose the age of twelve years old based on the common age in the Netherlands to start reproductive and sexual education and activities [42,43].

#### Inclusion criteria

Participants are adolescents in the age of 12–25 years old, exposed to family violence at home. Any adolescent encountering family violence at home, whether this is direct or indirect, is considered to be exposed to family violence.

#### Exclusion criteria

Since the internet-based self-support method is in Dutch, participants who do not speak the Dutch language are excluded. If, during the intervention period, a participant proves to be not a member of the target group, he/she is asked about the reason for his/her participation. The community manager will discuss this reasons anonymously with the supervising research team and decides if the participant is permitted to continue or will be excluded from further participation. In either case, his/her data are not being used in the evaluation.

#### Recruitment and informed consent

Participants will be recruited both offline and online, by posters and flyers of the website “Feel the ViBe” spread amongst stakeholders in the field, general websites and social media to reach as many participants as possible. In this target group, internet literacy is assumed.

According to the principles of Dutch law, for participants age 12 to 16 years old, both parents (or guardians) must consent in addition to the minor him/herself. However, in cases of family violence, informing the abusive partner can be potentially dangerous, for both the abused parent and the adolescent exposed to the violence, and safety cannot be ensured. Therefore, in these cases we ask informed consent from the abused parent, being the mother in most cases, only.

Participants register themselves by sending an email to the community manager through the contact form online. In this early phase, inquiring participants about the type and severity of the violence would enlarge the threshold for participation. We therefore choose to consider every potential participant eligible as target group. If participants have read the patient information letter, available on the homepage, and have no further questions, they are eligible to participate and will receive a login name and password. On their first login, participants have to give informed consent electronically. If applicable, participants are requested to provide contact information of their parent(s), who then receive a unique code and a link to give consent as well.

The intervention is internet-based without face-to-face components. This means that participants are quasi-anonymous for each other. Having multiple identities is being prevented by making the registration a manual process, including a IP-address check and e-mail contact, and the informed consent process.

### Settings

Participants can access “Feel the ViBe” from any computer, needing only their login name and password. All outcomes will be self-assessed through online questionnaires.

### Development of the intervention

Before starting the internet-based self-support method we conducted a literature research, consulted experts in the field and identified existing healthcare services available for adolescents exposed to family violence. We then identified three aims for a first version of the internet-based method “Feel the ViBe (Violence Beaten)”:

1. To offer peer support.

2. To offer information on family violence in broad sense, including information on dating violence and sexual and reproductive health.

3. To lower the threshold to existing healthcare by supplying information about healthcare services.

We set up criteria for the company building the internet-based self-support method, being:

1. Experience in building psychosocial interventions

2. Experience in building internet-based methods for adolescents

3. Employing a medical ICT specialist

4. Being able to comply to the safety and privacy rules for websites processing patient details.

5. Having all the necessary facilities, without having to hire consultants.

Two companies fitted these requirements, from which we selected the company that could provide personal support to the researchers daily, being “Re:publik”.

Based on expert opinions, literature, and in close collaboration with the medical ICT specialist from “Re:publik” we identified key elements and requirements of the internet-based method, divided in content available for everyone visiting “Feel the ViBe” and content for participants only. While a basic version of “Feel the ViBe” was being built, the researchers recruited GP’s to identify adolescents exposed to family violence who were not in an immediate need for professional care, and asked them to take part in a semi-structured interview about online support, reproductive and sexual health and wishes, needs, and expectations for general healthcare. Themes emerging from these interviews were used to formulate important features and subjects for the website and were added to the basic version of “Feel the ViBe”.

To improve the intervention further, we asked all participants who took part in the semi-structured interview to visit the website extensively as if they were actively searching for help at that time and comment on the website using a Web Evaluation Questionnaire (WEQ) about the goals, content and lay-out. Their were used to make final improvements on “Feel the ViBe”. This led to the intervention as described below.

### Intervention

“Feel the ViBe” is an internet-based intervention without face-to-face elements. “Feel the ViBe” is based on the theory that self-help, by means of peer-support and information, is an effective way of healthcare for the majority of the target group, while a minority will need further, face-to-face, help. The intervention is available online via http://www.feel-the-vibe.nl and consists of several elements, being amongst others a forum, a chat function, information, and a “ask the expert” function (Table 1). More information can be obtained by contacting the corresponding author. During the RCT the intervention will be ‘frozen’: no changes will be made to the intervention, except for changes due to unexpected events, such as bug fixes. These and other unexpected events, such as system downtimes, will be registered.

**Table 1:** "Feel the ViBe" elements

**Element**	**Extra information**	**Restrictions**
General information on exposure to family violence	Information by age (under-twelve, 12–17, 18–25 and parents) and by subject.	No restrictions
Research information & disclaimer	Information for participants and parents about research, safety and privacy.	No restrictions
Information on sponsoring	Homepage, bottom left.	No restrictions
Contact page	Option to register or ask questions to the community manager or researchers.	No restrictions
News page	Twitter newsfeed included. The news page states important information for participants such as major bug fixes, changes in content and scheduled maintenance.	No restrictions
Emergency exit	A button on every page directing participants to http://www.google.nl.	No restrictions
Electronic consent for participants	Consent is necessary to get access to other elements behind login	Available after first login. *Element will be removed after trial.*
Electronic consent for parents	Consent is necessary for participants under 16 years old to get access to other elements behind login	Accessible by e-mail link with a code. *Element will be removed after trial.*
Questionnaires	Measure outcomes and need to be filled out before accessing the elements behind login. Questionnaires will be activated in the personal menu. Questionnaires can be filled out one-by-one. Whenever possible, adaptive questioning is being used to make the burden as low as possible. There is a maximum of 15 questions per page. All items need to be filled out to submit a questionnaire. Participants cannot review their answers.	Available after first login, re-activated after 6,12, 18 and 24 weeks. *Element will no longer be obligated after trial.*
Personal menu	Menu for the participants with overview to all the available elements, access to the participants profile, digital testament, research information and contact information.	Login needed
User profile	The profile contains information on the participant, being: full name, nickname, avatar, sex, age, contact details and contact person. Only the nickname is available for other participants. The participant can choose a theme for the lay-out.	Login needed
Digital testament	The digital testament is required to fill out and lets participants choose how their data must be handled if they stop their participation.	Login needed
Ask the expert	Option to ask questions by e-mail to several experts, including a general practitioner, a sexologist, a psychologist and an expert in the field of family violence. Participants can also contact the community manager for general questions and questions regarding regular healthcare services. Response is given within 72 hours. During the RCT, this is the only available element for participants in the control group.	Login needed & questionnaires finished
Forum	The forum is meant to stimulate peer support. The community manager moderates the forum and stimulates contact. She will not intervene in the conversations except in case of offensive or factually wrong comments.	Login needed & questionnaires finished
Chat	Every two weeks we will offer a chat session for the participants with a specific theme and supported by an expert. Every other week there will be an unguided chat.	Login needed & questionnaires finished
Information	Information will be offered to the participants. Depending on the age in the profile, participants have access to tailored information about partner violence, sexual health, reproductive health, relations and healthcare. Participants are encouraged to discuss topics on the forum.	Login needed & questionnaires finished
Routine Outcome Measurement (ROM)	Directly after login and after logout a popup appears to the participant requesting to state their mood by means of smiley’s. The smiley is optionally visible in the profile as avatar.	Login needed
Facts & Figures	In a twelve-week cycle, participants receive a one-sentence fact of figure about family violence, sexual health, or reproductive health every day on their mobile or by e-mail.	Questionnaires finished

Overview of the elements of the internet-based self-support method “Feel the ViBe”, including restrictions for participants.

**Figure 1:**
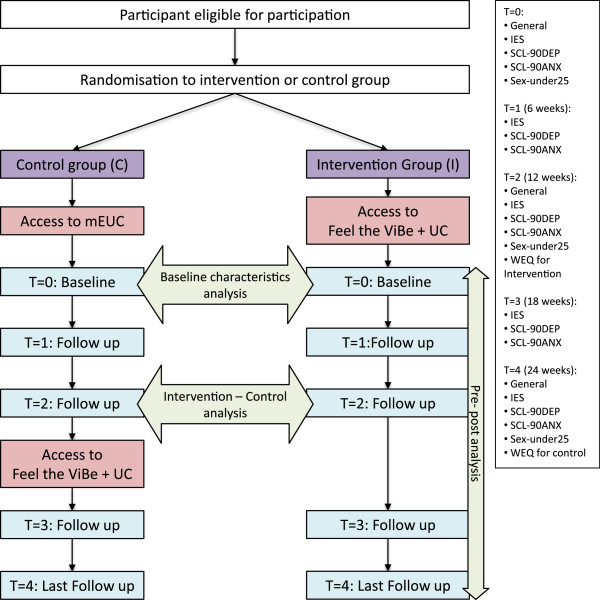
**Flowchart randomization procedure, received care and data collection.** Abbreviations: mEUC = minimally enhanced usual care, UC = usual care.

“Feel the ViBe” is to be used ad libitum. However, to stimulate participation, participants receive a fact or figure by email or text message every day (Table 1). Elements, such as the guided chat and “facts and figures”, are repeated every twelve weeks. Strictly spoken there is no hard endpoint to the intervention, because “Feel the ViBe” is meant to support participants as long as they need this. However, we decided to link measurements to this twelve week period (Figure 1). Some items are obligatory to fill out to gain access to other features, such as the questionnaires. Restrictions are given in Table 1. “Feel the ViBe” is monitored by a community manager who answers questions, guides the chat, moderates the forum and addresses possible safety issues with participants. To prevent bias, the Community Manager uses a protocol. Every action from the community manager not covered by the protocol will be registered, discussed with the supervising research team, and, if necessary, added to the protocol.

The intervention group will receive immediate access to “Feel the ViBe” after registration. Considering ethical concerns of withholding an intervention with a possible positive effect on physical and mental health, the control group will be a waiting list condition receiving minimally Enhanced Usual Care (mEUC) for twelve weeks before getting access to the intervention. mEUC is in general defined as non-study care enhanced in minor ways to address methodological or ethical issues. For this RCT mEUC is defined as Usual Care and access to a restricted version of “Feel the ViBe” offering only access to the user profile, consent forms, digital testament, research information, questionnaires and the “ask the expert” function to ask questions in case of emergency. Questions asked by members of the control group will not be answered by the Community Manager, but by one of the members of the Research Team who is not actively involved in the intervention, being author SLFW. If help is needed immediately, the participant will be referred to his/her GP (Figure 1).

### Outcomes

The intervention aims to offer peer support, information about family violence and sexual and reproductive health, including relational health, and mental support, in order to reduce PTSD symptoms and improve symptoms of depression and anxiety. Therefore we chose the outcomes measures as follows:

#### Primary outcome measure

•The Impact of Event Scale (IES) will be used to measure PTSD symptoms. The IES is a short set of 15 questions measuring the impact of events and the amount of distress associated with events. It comprises the subscales Intrusion (8 items, mean α = 0.86) and Avoidance (7 items, mean α = 0.82). The IES is measured at t = 0, t = 1, t = 2, t = 3 and t = 4 for both groups [44-46].

•The Depression and Anxiety subscales of the Symptom CheckList-90-R (SCL-90-R DEP and ANX) will be used to measure an improvement in symptoms of depression and anxiety. The SCL-90-R DEP and ANX subscales measure symptoms of depression and anxiety during the previous week on a five-point Likert scale. Both subscales showed good convergent and divergent validity, and high internal consistencies. The SCL-90-R is validated for participants of twelve years old and older. The depression subscale comprises 16 items (α = .90), the anxiety subscale comprises 10 items (α = .88). The SCL-90-R DEP and ANX subscales are measured at t = 0, t = 1, t = 2, t = 3 and t = 4 for both groups [47].

#### Secondary outcome measures

•Routine Outcome Measurement (ROM) will be used to follow participants from session to session. On every login, participants will see a pop-up asking them how they would grade their mood at that precise moment on a visual analogue scale using smiley’s. After logging out they will receive a pop-up asking the same question. The scores and differences in scores before and after the session are analyzed to find any direct effects of visiting the website [48].

•An adapted version of the “Seks onder je 25e” (Sex-under-25) questionnaire will be used to measure an increase in knowledge on sexual and reproductive health, including relational health and a decrease of sexual risk taking behaviour. This validated questionnaire is used in a survey in the Netherlands every few years amongst adolescents twelve to twenty-five. In 2011 the questionnaire was filled out online by a representative sample of 10.000 youngsters. It discusses sexual, reproductive and relational health in a broad way handling for example topics as sexual education, ‘the first time’, negative experiences, and contraception. Using this questionnaire will enable us to compare health status of the target group with a population sample. The “Seks onder je 25e” (Sex-under-25) questionnaire is measured at t = 0, t = 2 and t = 4 for both groups [49].

#### Process evaluation

•The web evaluation questionnaire (WEQ) will contain questions about content, layout, the perceived effectiveness and usefulness of the website, as well as the question to give the website a motivated overall score from 0 to 10, based on their experiences and taking into account their own wishes and needs. The evaluation questionnaire has as goal to identify issues for further improvement of “Feel the ViBe”, to collect possible facilitators and barriers for implementation and to evaluate if the website meets the expectations of the target group. The WEQ is measured twelve weeks after getting access to Feel the ViBe, being t = 2 for the intervention group and t = 4 for the control group.

•“Use” is measured by the collection of quantitative data - being amongst others and duration, visited pages, and visitor numbers - and qualitative data - being forum and chat entries and questions asked to the experts – and is monitored on a continuous base [50,51].

•Demographic variables and data on other (health) care and support received will be collected. At t = 0, participants will also be asked to their expectations, needs and wishes for “Feel the ViBe”.

•After participants have finished all components of the intervention, including all questionnaires, they will be asked if they consent to take part in an interview to discuss in dept their experiences with the intervention.

All questionnaires are provided online and are mandatory to get access to other features. The IES, the SCL-90 and the “Sex-under-25” questionnaire have been administered online before in other studies. Both the general questionnaire and the WEQ have been developed for this trial. Comments and suggestions of test users were incorporated. All the questionnaires have been tested for usability and technical functionality. All responses are being captured automatically.

The primary outcome measures, being the IES and the SCL-90R subscales will be administered four times. This will give us the possibility to determine the optimal duration of the intervention. Participants receive a reminder in their personal menu and via a text message (Figure 1).

### Sample size

For the calculation of de sample size of the RCT, we searched for studies investigating internet-based methods with the Impact of Event Scale (IES) as a primary outcome measurement [52-55]. We set the confidence interval to 95% and the power to 80%. We calculated the minimal sample sizes using the Cohen’s *d* effect sizes found in these studies. In general, an effect of 0.5 is considered to be a large effect. In the four selected studies, the effect size ranged from 0.7 (leading to 26 participants needed) to 2.0 (4 participants needed), with a mean of 11 and a median of 9 participants needed for each group. Considering the relatively high effect sizes found in these studies and because both the subject of the study and the age of the participants can lead to high drop-out, as seen before in a group of sexually abused adolescents, we aim to include 50 participants for each group at t = 0 [53].

### Randomization

Randomized trials with children and adolescents are controversial. The subject of our study enhances this even more. However to measure the effectiveness of an intervention, a randomized controlled trial is preferred above pre- post-test designs. Participants registering themselves online will be randomized to the intervention or the control group using the method of Minimization, as described by Taves (1974) [40,41]. Subcategories will be sex (male or female) and age (12–17 years old and 18–25 years old). This method minimizes the difference between the intervention and the control group. The allocation is carried out with help of a computer program by a research assistant.

Due to the nature of the study, participants cannot be blinded. Because of the nature of the overall study, containing both quantitative and qualitative parts, blinding of the investigators is difficult because the researchers need to know the group of the participant to be able to perform qualitative analysis and to follow each participant individually for the process evaluation. However questionnaires will be collected automatically during the trial in a SPSS file and delivered anonymized to the researchers.

### Data analysis

Data will be collected for the duration of one year.

First, descriptive statistics of the characteristics of the intervention and the control group will be compared to check whether randomization resulted in similar groups.

The effectiveness of the intervention will be tested with ANOVA of the primary outcome measures. This gives us the opportunity to compare not only the intervention with mEUC (between groups effects), but also to do pre-posttest analysis for all participants (within subjects effects). Data will be analyzed in SPSS. Effect sizes will be expressed in Cohen’s *d* to make data comparable with earlier studies. Data will be analyzed both together and separately for the group 12–17 years old and 18–25 years old.

In e-health trials attrition is typically high and not all participants will use the intervention as intended. A participant will be excluded from analysis if he/she visited the intervention less than five times. His/her entry data (t = 0) will be used to describe baseline characteristics [56].

To interpret the findings, usage data, the WEQ, and the interviews held with the participants after finishing their participation will be analyzed alongside. Qualitative data will be analyzed with qualitative coding [57]. Interview data will be recorded and transcribed. Two researchers will study all transcripts independently, identify themes and establish the definite codes. Consensus will be reached in mutual discussion. Subsequently these outcomes will be formulated and interpreted in the supervising research group for final results. Quotes will be used to underline the results.

### Ethical and safety issues

The Committee on Research Involving Human Subjects of the Radboud University Nijmegen Medical Centre (Dutch initials: CMO) has assessed this study and judged that the study does not fall within the remit of the Medical Research Involving Human Subjects Act (WMO). Therefore, the study can be carried out (in the Netherlands) without approval by an accredited research ethics committee (2011/053. NL nr 35813.091.11. March, 16th, 2012).

After signing up, all participants receive a username and password to create security and to follow them for analyzing properties. Each participant will receive a number. We hereby secure the anonymity of adolescents. To enhance the safety even more, the website is based on a secured server, which meets the safety criteria for e-health applications containing medical information. Acquired data will be handled according to the digital testament of the participant. Quantitative data will be collected during the trial on a secured server. The community manager moderates the interactive parts of the website and, if applicable, asks participants to remove personal information.

Participants can email or call the community manager, even if they have not completed the obligated parts. Emails are answered within 72 hours. If there are concerns, for the safety of a participant or his/her close family, the participant will be addressed by the community manager to discuss this further. All participants are asked to give contact details of an adult they trust. In case of severe danger, the community manager can contact this person with consent of the participant, or, if a participant is below 16, also without consent. The participant is free to decide whether he/she continues with Feel the ViBe.

## Discussion

This study protocol describes the process of developing and evaluating an internet-based self-support method “Feel the ViBe” for children, adolescents and young adults exposed to family violence.

E-health research is still relatively new and has some limitations due to its online nature: attrition is usually high which makes sample size calculations difficult. Data must be handled differently than offline data, because safety and privacy issues are a concern, especially in our target group. While making this protocol, the authors followed the CONSORT-EHEALTH checklist version 1.6.1 and The Checklist for Reporting Results of Internet E-surveys (CHERRIES) [58-61].

Publishing a research protocol before starting a RCT is essential. It not only prevents publication of positive-only results, by changing the protocol or outcome-measures, it also enables researchers to discuss their intervention and methods more in-depth. There are some limitations to this study: developing an online intervention for a vulnerable group of adolescents means it is hard to reach a representative sample. For this RCT, participants will register themselves, which means only participants actively searching for help and having access to the internet will be included. However, in the Netherlands, 93% of children above twelve years old have access to the internet and the researchers try to further minimize this bias by advertising the intervention in a broad way, both online and offline [32,33]. To keep attrition to a minimum, researchers send reminders for questionnaires and daily “facts and figures” to the participants. A process evaluation is being held to investigate, amongst others, recruitment and attrition. Lastly, the community manager monitors the intervention to motivate participants to be actively involved. To minimize possible bias, all actions of the external community manager will be registered and discussed with the supervising research team. Results of the questionnaires will be collected during the trial and delivered to the researchers at the end of the trial. In this way, the community manager cannot be influenced by questionnaire results.

“Feel the ViBe” is not the first intervention for this target group. However, “Feel the ViBe” is an online-only intervention, it offers help to older children, adolescents and young adults and is self-supporting. The intervention “Feel the ViBe” is developed in close collaboration with the target group. A RCT and a process evaluation will follow to test effectiveness and help us understand how the effects of the intervention can be explained, how the interventions meet the expectation of the participants and what possible barriers and facilitators for implementation are. Qualitative analysis will complete quantitative data. This makes “Feel the ViBe” unique in its field.

## Abbreviations

Feel the ViBe: Feel the violence beaten; UC: Usual care; mEUC: minimally Enhanced Usual Care; WEQ: Web evaluation questionnaire; PTSD: Post traumatic stress disorder; STD: Sexually transmitted disease; IES: Impact of event scale; SCL-90-R DEP: Symptom checkList-90-R, depression subscale; SCL-90-R ANX: Symptom checkList-90-R, anxiety subscale; ROM: Routine outcome monitoring; CHERRIES: The CHEcklist for reporting results of internet E-surveys.

## Competing interests

Dr Lo Fo Wong reported receiving governmental and non-profit grants for a research project on mentor mother help for interpersonal violence; working as a trainer on recognizing partner violence; and receiving travel expenses to a WHO consultation group meeting on violence against women. Prof. Lagro-Janssen reported receiving governmental and non-profit grants to study partner violence. Prof. Prins reported receiving grants to study e-health interventions for patients with cancer.

## Authors’ contributions

SLFW, JP, ALJ and KRN are responsible for the design of the study. MV participated in the design of the study. KRN wrote the first draft of the manuscript and SLFW, JP, and ALJ revised the manuscript critically. All authors read and approved the final manuscript.

## Authors’ informations

KRN is a family physician trainee and PhD student at the department Gender & Women’s Health, Primary and Community Care, Radboud University Nijmegen Medical Centre, the Netherlands. JP is an clinical psychologist and academic professor Medical Psychology at the Radboud University Nijmegen Medical Centre, the Netherlands. SLFW is a family physician and senior researcher at the department Gender & Women’s Health, Primary and Community Care, Radboud University Nijmegen Medical Centre, the Netherlands. MV is a physician and sexologist at the department of Gynaecology and Obstetrics at the Radboud University Nijmegen Medical Centre, the Netherlands. ALJ is a family physician and academic professor Gender & Women’s Health at the Radboud University Nijmegen Medical Centre, the Netherlands.

## Pre-publication history

The pre-publication history for this paper can be accessed here:

http://www.biomedcentral.com/1471-2458/13/226/prepub
